# Expanding the Family of Heterostructured CdSe/CdS Core/Crown Nanoplatelets: Six- and Seven-Monolayers-Thick Species

**DOI:** 10.1021/acs.chemmater.5c00650

**Published:** 2025-08-07

**Authors:** Volodymyr Shamraienko, Valeriia Haidei, Artsiom Antanovich, René Hübner, Steven C. Erwin, Vladimir Lesnyak, Alexander Eychmüller

**Affiliations:** † Physical Chemistry, 9169 TU Dresden, Zellescher Weg 19, 01069 Dresden, Germany; ‡ Institute of Physical Chemistry and Electrochemistry, Leibniz University Hannover, Callinstraße 3A, 30167 Hannover, Germany; § Institute of Ion Beam Physics and Materials Research, Helmholtz-Zentrum Dresden-Rossendorf e.V., Bautzner Landstrasse 400, 01328 Dresden, Germany; ∥ Center for Computational Materials Science, Naval Research Laboratory, Washington, District of Columbia 20375, United States

## Abstract

The growth of atomically flat CdSe nanoplatelets (NPLs) thicker than 5 monolayers (ML) remains a major challenge in colloidal semiconductor synthesis, particularly for core/crown heterostructures. Here we report the successful synthesis of zinc-blende CdSe NPLs with unprecedented thicknesses of 6 and 7 ML, exhibiting sharp photoluminescence at 579 and 596 nm, respectively. We demonstrate that these thick NPLs can serve as cores for CdSe/CdS core/crown heterostructures, confirmed by lateral size expansion and the emergence of characteristic CdS absorption features. Through density-functional theory calculations, we uncover a critical relationship between NPL shape and crown growth: fluoride species bind three times stronger to {100} facets of rectangular NPLs compared to {110} facets of square NPLs, effectively poisoning crown growth on rectangular species. This mechanistic insight explains the shape-dependent success of crown formation and provides a framework for controlling two-dimensional (2D) semiconductor growth. Our optimized synthesis of thick core NPLs and their crown-enhanced derivatives significantly expands the spectral range of CdSe-based NPLs, advancing their development as narrow-line width red emitters.

## Graphical abstract

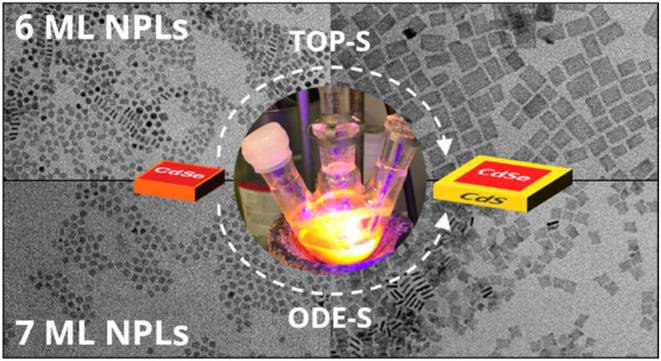

## Introduction

Colloidal two-dimensional (2D) zinc blende CdSe nanocrystals have emerged as a material of interest for researchers in various fields due to their atomically precise thickness, reflected in well-defined optical properties through strong one-dimensional quantum confinement, which enables their potential application in optoelectronic devices, and their high surface-to-volume ratio, which facilitates application in chemical sensing.^1–8^ The transformation of these materials by doping with different elements, shell growth across the structure, cation exchange reactions, and the synthesis of multicomponent heterostructured nanoplatelets (NPLs) further stimulates research into these materials, as these modifications open new potential avenues for their applications.^9–18^ The 3–5-monolayer (ML)-thick CdSe NPLs, which are the most studied to date, are generally formed by the addition of short-chain carboxylate ligands to a hot mixture of cadmium and selenium precursors in octadecene (ODE).^19–21^ Understanding the selective binding of ligands to the facets of CdSe NPLs not only facilitates their further postsynthetic lateral-seeded growth, but also enables the preparation of various core/crown NPLs such as CdSe/CdS, CdSe/CdTe, CdSe/CdSe_1–*x*_Te_*x*_, and various other combinations which, however, are still limited to the family of 3-to-5-ML CdSe NPLs.^22–27^

The direct synthesis of CdSe NPLs with more than 6 atomic layers of cadmium and 5 layers of selenium remains a challenge. However, several promising synthesis methods have been developed. For example, Cho et al. presented the preparation of 6-ML CdSe NPLs by combining the conventional platelet synthesis with the addition of metal chlorides that release chloride anions in situ,^28^ while Meerbach et al. used a combination of highly reactive cadmium nanoparticles and small fluoride anions, resulting in NPLs with the same thickness.^29^ Controlling the thickness of the NPLs studied by Christodoulou et al. enabled the synthesis of 6-, 7-, and 8-MLs-thick NPLs through a two-step reaction, in which the addition of cadmium chloride switches the growth from 2D to three-dimensional (3D).^30^ Despite all these advances, colloidal atomic layer deposition (c-ALD) and dissolution/recrystallization are still the methods of choice for the synthesis of thick cadmium chalcogenide NPLs, even though they suffer from numerous defects and poor optical properties and are not suitable for further lateral growth or heterostructure formation.^31–33^ At the same time, the subsequent modification of the NPLs by shell or crown growth is the most easily accessible method for improving and tuning their properties. Similar to quantum dots, the continuous growth of a CdS or ZnCdS shell over the entire surface of CdSe NPLs at high temperatures can lead to an increase in photoluminescence quantum yield (PL QY) and optical stability by passivating surface defects.^34–36^ Depending on the choice of shell composition and shell thickness, various changes to the NPL properties can be achieved, which further broaden the application possibilities.^37–40^ Crown growth, on the other hand, focuses on the passivation of defects at the edges of the NPLs, whereby the thickness of the core/crown heterostructure remains the same as for the core NPLs and only leads to an increase in the lateral dimensions.^23^ For instance, passivation of the edge defects with CdS can improve the optical properties without significantly altering the position of absorption and PL spectra of the core, while the shell growth leads to a red shift and broadening of the spectra. If desired, a change in emission position can be achieved by varying the crown composition (e.g., CdTe, CdSTe, CdSeTe),^24,25,41^ which can result in type-II heterostructures.

Here, we present the direct synthesis of laterally rather monodisperse (approximately 8 nm) round-edged 6- and 7-ML CdSe NPLs with PL maxima at 579 and 596 nm followed by overgrowing them with CdS to form core/crown CdSe/CdS heterostructures. Our studies show that although the addition of metal fluoride (or more general metal halides) is necessary for the successful synthesis of thicker NPLs, it does not play a crucial role in the further growth of the CdS crown per se. Theoretical calculations revealed that the fluoride poisoning of square NPLs (with {110} edges) is much lower than that of rectangular (with {100} edges) ones. This finding opens an exciting perspective on the formation of heterostructures, however, it does not explain why a core/crown structure was achieved in this work, since HRTEM analysis revealed that square, rectangular, and CdSe/CdS core/crown NPL all have {100} edges. The 6-ML CdSe/CdS core/crown NPLs grown up to 20 nm × 40 nm show a PL maximum at 591 nm with a PL QY of 42%. The methods used for 7-ML CdSe NPLs allow us to grow the CdS crown up to 10 nm × 15 nm with a PL maximum at 608 nm and a PLQY of 72%. We believe these methods represent a significant step toward the precise colloidal synthesis of even thicker 2D CdX (X = S, Se, Te) NPLs and the preparation of their core/crown heterostructures, further pushing the optical properties of narrow and efficient emitters to the longer wavelengths.

## Experimental Section

### Chemicals

Cadmium acetate (Cd­(OAc)_2_, 99.995%), cadmium acetate dihydrate (Cd­(OAc)_2_·2H_2_O, for analysis), cadmium oxide (CdO, 99.99%), zinc acetate dihydrate (Zn­(OAc)_2_·2H_2_O, 99%), myristic acid (HMyr, ≥99%), oleic acid (OlAc, 90%), 2-ethylhexanoic acid (≥99%), tetramethylammonium hydroxide pentahydrate (TMAH, ≥97%), sulfur (S, powder, 99.98%), selenium dioxide (SeO_2_, 99.8%), chloroform (CHCl_3_, ≥99%), and cadmium chloride (CdCl_2_, 99.99%) were purchased from Merck. Selenium powder (Se, 160 mesh, 99.99%) was purchased from Chempur. Cadmium fluoride (CdF_2_, 99%), tri-*n*-octylphosphine (TOP, 97%), and cadmium bromide (CdBr_2_, 99%) were purchased from ABCR. Methanol (HPLC grade) and *n*-hexane (97%) were purchased from VWR Chemicals. Ethanol (EtOH, 99.8%) was purchased from Fisher Chemical. Tetradecanoic acid (98%) was purchased from Alfa Aesar. All reagents were used as received without further purification.

### Cadmium Myristate (CdMyr_2_) Preparation

Cadmium myristate was prepared according to the procedure published by Mitrofanov et al.^10^ In a 300 mL conical flask, 200 mL of methanol, 4.56 g (20 mmol) of tetradecanoic acid, and 3.62 g (20 mmol) of TMAH were mixed until completely dissolved, followed by the dropwise addition of the solution of 2.66 g (10 mmol) of Cd­(OAc)_2_·2H_2_O in 64 mL of methanol upon vigorous stirring. The resulting product was washed with methanol by centrifugation 6 times at 3824 RCF. The final precipitate was dried in vacuum.

### Synthesis of 6-ML CdSe NPLs

In a three-neck 50 mL flask, 300 mg (0.53 mmol) of CdMyr_2_, 100 mg (0.44 mmol) of HMyr, and 28 mL of ODE were mixed and degassed at 80 °C for 1 h upon vigorous stirring. Subsequently, the flask was filled with argon, heated to 310 °C and kept at this temperature until the mixture started to turn gray and turbid. Directly after this color change, the flask was rapidly cooled down to room temperature with a cold water bath and degassed again for 30 min, followed by filling with argon and heating up to 280 °C. When the solution reached 260 °C, a dispersion of 24 mg (0.3 mmol) of Se in 2 mL of ODE (prepared in a nitrogen-filled glovebox and sonicated for 30 min) was quickly injected. After 60 s, 81 mg (0.54 mmol) of CdF_2_ were added (when the same molar amounts of CdCl_2_ and CdBr_2_ were used, no NPLs were formed), and 45 s later, 140 mg (0.61 mmol) of Cd­(OAc)_2_ were introduced into the reaction mixture. After 40 min at 280 °C, the heating mantle was removed, and during the cooling process with the water bath at 180 °C, 3 mL of OlAc were injected. The as-synthesized NPLs were centrifuged at 10621 RCF for 3 min. The supernatant was discarded, and the precipitate was redispersed in 25 mL of hexane and 5 mL of EtOH and sonicated for 5 min. The resulting solution was centrifuged at 10621 RCF for 3 min. The resulting supernatant was mixed with 7 mL of EtOH and centrifuged again for 3 min at the same speed. Further purification of the precipitate containing 6-ML NPLs was carried out by size-selective precipitation with hexane and EtOH. The resulting particles were dispersed in 3 mL of CHCl_3_ for further analysis or in ODE for CdS crown growth.

### Synthesis of 6-ML CdSe/CdS Core/Crown NPLs (20 nm × 40 nm)

In a three-neck 25 mL flask, 96 mg (0.42 mmol) of Cd­(OAc)_2_, 180 μL of OlAc, and a dispersion of 6-ML CdSe core NPLs (synthesized as described above) in 10 mL of ODE were mixed and degassed at room temperature for 30 min. The flask was then purged with argon, and the temperature was set to 250 °C. During the heating, at 215 °C, an addition of 250 μL of 1 M S-in-TOP mixed with 3 mL of ODE was started via a syringe pump at a rate of 6 mL/h. After the S-precursor addition, the reaction mixture was left to react for 5 min and subsequently cooled down to room temperature with the water bath. The resulting product was mixed with 5 mL of EtOH and centrifuged for 3 min at 10621 RCF. The supernatant was redispersed in 10 mL of hexane, shaken for 30 min, and centrifuged again for 2 min at 6797 RCF. This procedure was repeated until the complete removal of byproducts (large 3-ML CdS NPLs). The resulting supernatant, containing the 6-ML CdSe/CdS core/crown NPLs, was purified by size-selective precipitation with hexane and EtOH. The 10 nm × 15 nm core/crown NPLs were synthesized using the same method with reducing 1 M S-in-TOP volume to 125 μL and increasing the injection speed to 12 mL/h; for 15 nm × 20 nm NPLs we used the same reduced amount of S-in-TOP but a slower injection rate of 6 mL/h.

### Synthesis of 7-ML CdSe NPLs

A mixture of 70 mg (0.54 mmol) of CdO, 340 mg (1.49 mmol) of HMyr, and 28 mL of ODE in a 50 mL three-neck flask was heated to 100 °C under vacuum for 30 min. The reaction flask was then purged with argon and heated to 310 °C. During the heating process, at ca. 280 °C, the reaction mixture turned colorless and then, at 310 °C, started to turn white turbid and later gray/brown turbid, at which point the flask was rapidly cooled down with the water bath to 100 °C and degassed again for 30 min. The evacuated reaction flask was filled with argon and heated up to 280 °C. During heating, upon reaching 260 °C, a dispersion of 24 mg (0.3 mmol) of Se in 2 mL of ODE (prepared in the glovebox and sonicated for 30 min) was quickly injected, after 45 s, 81 mg (0.54 mmol) of CdF_2_ were added, and 30 s later, 140 mg (0.61 mmol) of Cd­(OAc)_2_ were introduced into the reaction mixture. Directly after the addition of Cd­(OAc)_2_, an addition of a previously prepared deep orange solution of 54 mg (0.49 mmol) of SeO_2_ in 6 mL of ODE (by heating to 210 °C and stirring until complete dissolution of SeO_2_) was started via a syringe pump at a rate of 16 mL/h. Directly after the Se-precursor addition, the heating mantle was removed, and after the reaction solution reached 180 °C, 3 mL of OlAc were added. The resulting particles were purified using the same method described for the 6-ML NPLs.

### Synthesis of 7-ML CdSe/CdS Core/Crown NPLs

50 mg (0.23 mmol) of Zn­(OAc)_2_·2H_2_O, 100 μL of OlAc, and a dispersion of 7-ML CdSe core NPLs (synthesized as described above) in 5 mL of ODE were mixed in a 25 mL three-neck flask and degassed at room temperature for 30 min. Subsequently, the flask was filled with argon and heated up to 215 °C. After the desired temperature was reached, 0.4 mL of a cadmium precursor (26 mg (0.2 mmol) of CdO, 200 μL of 2-ethylhexanoic acid mixed with 200 μL of ODE heated to 230 °C until completely dissolved, cooled to room temperature naturally, and purged with nitrogen) and 1 mL of a sulfur precursor (250 μL of 1 M S-in-ODE freshly prepared and heated in a nitrogen-filled glovebox until the sulfur has completely dissolved and mixed directly with 1 mL of ODE) were mixed together and added into the reaction mixture from a syringe pump at a rate of 6 mL/h. After the addition, the reaction mixture was rapidly cooled down to room temperature with the water bath. The resulting particles were purified using the same methods described for the 6-ML CdSe/CdS NPLs. However, the shaking step is not necessary, as no large CdS NPLs are formed.

### Characterization

#### Optical Characterization

Absorption spectra were recorded using an ultraviolet-visible-near infrared (UV–vis–NIR) spectrophotometer Cary 5000 (Varian). PL and PL excitation (PLE) spectra were recorded with a FluoroMax-4 spectrofluorometer (Horiba Jobin Yvon Inc.). Time-resolved PL measurements were conducted at room temperature using a Horiba Jobin Yvon FluoroCube-01-NL under excitation with a NanoLED-350 pulsed laser diode (λ = 349 nm, pulse duration <1 ns). Average PL lifetimes were calculated when the initial signal intensity was reduced to 10,000 counts e^–1^. To evaluate the absolute PLQYs, we used a FluoroLog-3 spectrofluorometer (Horiba Jobin Yvon Inc.) equipped with a Quanta-φ integrating sphere.

#### Electron Microscopy

Bright-field transmission electron microscopy (TEM) imaging was performed on a JEOL JEM-1400 Plus microscope operated at 120 kV. High-angle annular dark-field scanning TEM (HAADF-STEM) imaging and spectrum imaging analysis based on energy-dispersive X-ray spectroscopy (EDXS) were performed at 200 kV with a Talos F200X microscope equipped with an X-FEG electron source and a Super-X EDX detector system (FEI). Prior to the STEM analysis, the specimen mounted on a high-visibility low-background holder was placed for 2 s into a model 1020 Plasma Cleaner (Fischione) to remove potential contamination. Powder X-ray diffraction (XRD) analysis was performed on a Bruker AXS D2 Phaser instrument with a LYNXEYE/SSD160 detector in Bragg–Brentano geometry. The samples were dropcast from concentrated dispersions on a single-crystalline silicon wafer. Cu K_α1_ (l = 1.54056 Å) radiation, a current of 10 mA, and an operation voltage of 30 kV were used.

## Results and Discussion

The procedure for the synthesis of 6- and 7-ML thick CdSe NPLs is based on the approach published by Meerbach et al.^29^ and described in detail in the Experimental Section. The major improvement in our method involves the successive addition of a selenium dispersion in ODE, CdF_2_, and Cd­(OAc)_2_ into a hot colloidal solution of in situ-formed metallic Cd nanoparticles. Rapid injection of the selenium precursor at 260 °C initiates the formation of CdSe nuclei, likely in the form of stable, magic-sized nanoclusters, which grow further upon the addition of CdF_2_. The addition of cadmium halides indeed leads to a size increase of the clusters, as shown in a recent study by Mazzoti et al.^42^ Despite the fact that chloride and bromide anions reduce the surface energy and consequently accelerate the growth of magic-sized clusters much more efficiently than fluoride, we suspect that the solubility of CdCl_2_ and CdBr_2_ (the same molar amount as of CdF_2_) at a given temperature is too high for the synthesis investigated, since no NPLs were formed by using these Cd halides (Figure S1a,b in the Supporting Information). We assume that rapid dissolution not only releases large quantities of the halide ions required for the growth but also swiftly increases the amount of cadmium precursor in the reaction mixture, which can shift the delicate equilibrium between the precursors and the clusters after selenium injection required for the successful synthesis of 6- and 7-ML NPLs. On the other hand, high thermal energy in combination with reduced surface energy (mediated by halides) can lead to the undesired isotropic growth of the particles, resulting in CdSe quantum dots. We hypothesize that in our case a prolonged dissolution of cadmium fluoride leads to a slow release of fluorine active species, which slightly reduce the surface energy of the formed clusters and, in combination with the thermal energy, facilitate the growth of the crystals in a more controlled manner. At the same time, a slow increase of the amount of the cadmium precursor does not lead to significant changes in the already existing equilibrium between the precursors. The further addition of Cd­(OAc)_2_ 45 s later promotes the 2D growth and leads to the successful synthesis of 6-ML CdSe NPLs.

It is important to note that the addition of Cd­(OAc)_2_·2H_2_O does not result in the successful synthesis of 6-ML platelets. We assume this is related to changes in the salt’s melting point and decomposition temperature. The dehydration temperature of Cd­(OAc)_2_·2H_2_O is between 89 and 150 °C.^43,44^ When this salt is added at the reaction temperature (260 °C) it immediately causes a sharp increase in the concentration of the cadmium precursor and acetate ions. Additionally, the released water molecules may hydrolyze the surface acetate, resulting in the release of acetate ions at 200 °C, which then react.^43^ This rapid increase in precursor concentration, combined with the selenium precursor and acetate anions, likely leads to the formation of thinner platelets (3-, 4-, or 5-ML) along with various byproducts. In contrast, the melting and decomposition point of anhydrous Cd­(OAc)_2_ is between 250 and 280 °C.^44^ When added to the reaction mixture, this salt melts and dissolves slowly, ensuring a more controlled release of acetate and cadmium ions. This gradual release facilitates the growth of 6-ML CdSe NPLs without significantly disrupting the delicate equilibrium between the precursors.

TEM imaging of the NPLs revealed round-cornered species with lateral dimensions of approximately 8 ± 0.7 nm (Figure 1a). To increase the lateral size of the NPLs and to make their shape more defined, we attempted a postsynthetic seeded growth approach based on the work of Schlosser et al.^45^ Our method used the same protocol as for crown growth, but with 125 μL of 1 M Se-in-TOP instead of the S-precursor and a 6 mL/h injection rate. The resulting NPLs exhibited average lateral dimensions up to 10 nm × 13 nm and had a rectangular shape with sharp corners (Figure 1b). This increase in size can also be traced by optical spectroscopy (Figure 1c): the heavy-hole (hh) transition of the as-synthesized 6-ML NPLs was at 573 nm, while it shifted to 579 nm after the seeded growth, and their PL followed the same trend and shifted from 579 to 583 nm. These results are consistent with previous studies on the lateral, size-dependent adjustment of the optical properties of 2D CdSe NPLs.^20,46,47^ The initial 6-ML CdSe NPLs show a narrow PL with full width at half-maximum (FWHM) of 13 nm, which can be explained by strong lateral confinement regime in the small NPLs, and is consistent with previously published results,^29,47^ and explains the lower PLQY of 34%. The small lateral dimensions result in a higher proportion of defect-rich NPL edges relative to the total surface area. After lateral growth, the FWHM of the PL spectra decreased to 12 nm, consistent with weaker lateral confinement in larger NPLs, as reported in previous studies.^47^

**Figure 1:**
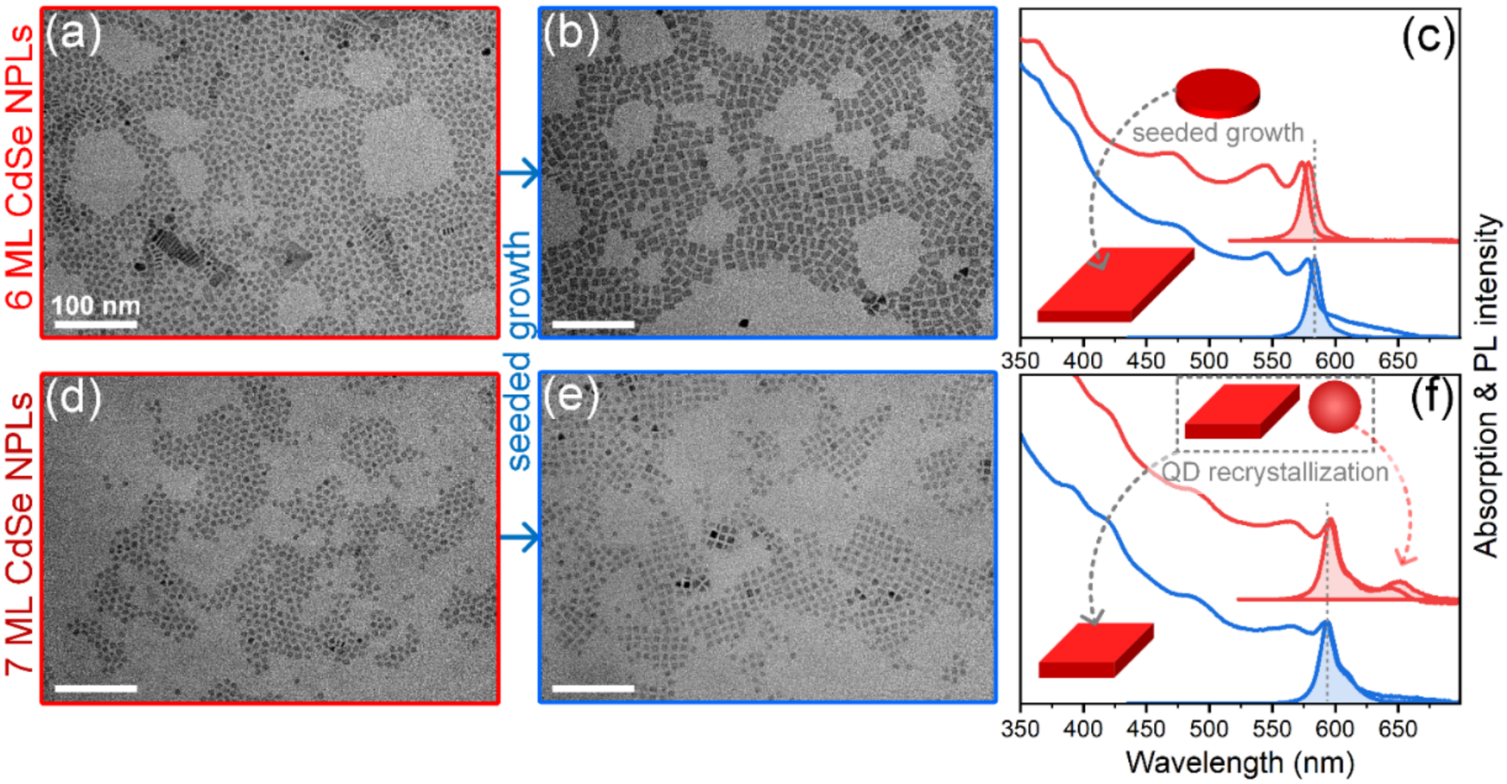
TEM images of 6-ML CdSe NPLs before (a) and after the seeded growth (b) with corresponding absorption and PL spectra (c). TEM images of 7-ML CdSe NPLs before (d) and after the attempted seeded growth (e) with corresponding absorption and PL spectra (f). Colors of the boxes around TEM images in (a–e) correspond to the colors of the spectra in (c, f).

The same method was used to grow thicker 7-ML CdSe NPLs, with the only significant modification being the controlled addition of SeO_2_ solution in ODE immediately after the addition of Cd­(OAc)_2_. We hypothesize that after the magic-sized cluster growth, the direct addition of the highly active Se-precursor leads to the additional nucleation of a 2D island. This results in cluster growth through an additional layer on one of the cluster facets, as recently demonstrated by the Norris group.^42^ Further costabilization with cadmium acetate/fluoride promotes the formation and growth of thicker 7-ML CdSe NPLs. TEM imaging of the product shows small platelets with lateral dimensions similar to those of their 6-ML counterparts (8 ± 0.51 nm) (Figure 1d). The 7-ML CdSe NPLs obtained in this way exhibit a remarkably high PLQY of 47%, demonstrating the efficient stabilization of these particles. However, similar to the previously discussed laterally small 6-ML NPLs, a stronger lateral quantum confinement leads to a broadening of the FWHM up to 15 nm.^47^ Interestingly, attempts to increase the lateral size of these platelets using the same approach as for the 6-ML NPLs yielded different results. The platelets became sharp-edged, but their lateral size unexpectedly decreased (Figure 1e). The heavy-hole (hh) transition, initially at 594 nm, shifted to 591 nm, and the PL peak shifted from 596 to 593 nm (Figure 1f). Notably, after seeded growth, CdSe quantum dots present in the as-synthesized 7 ML sample, as evidenced by a small PL peak at 650 nm (Figure 1f), disappeared, probably transforming into corresponding NPLs.

In the next step, we focused on the synthesis of core/crown CdSe/CdS NPL heterostructures, which exhibit improved optical properties compared to the core NPLs. The defect-rich edges of the core are fully passivated in the core/crown, ensuring higher stability and PLQY. To adjust the lateral dimensions of the CdS crown, we used different amounts of the S-precursor and varied its injection speed. According to TEM imaging, the growth of the CdS crown on 6-ML CdSe NPLs is evidenced by the gradual increase of lateral dimensions of the resulting core/crown NPLs to 10 nm × 15 nm (125 μL of 1 M S-in-TOP, 3 mL of ODE, 12 mL/h injection rate), 15 nm × 20 nm (125 μL of 1 M S-in-TOP, 3 mL of ODE, 6 mL/h injection rate), and 20 nm × 40 nm (250 μL of 1 M S-in-TOP, 3 mL of ODE, 6 mL/h injection rate) (Figure 2b). In all cases, crown growth transformed the NPL shape from disc-like to well-defined rectangles with sharp corners. This transformation was accompanied by significant changes in their absorption spectra (Figure 2c). As can be seen from the spectra, the hh transition in the absorption shifted to 586 nm after the growth of the largest CdS crown, while the corresponding PL peak shifted to 591 nm. These shifts can be attributed to changes in the dielectric environment surrounding the CdSe core and the overall increase in the lateral dimensions of the heterostructure, which result in a weaker lateral quantum confinement. The reduction of the PL FWHM to 11.4 nm supports this observation. Weakened quantum confinement in thicker NPLs also leads to a more pronounced delocalization of the electron wave function across the entire heterostructure, resulting in larger spectral shifts. The formation of CdS crown also contributes to a redshift in the optical properties by altering the dielectric constant.^48^ A new absorption feature at 447 nm, which becomes more prominent as the lateral size of the crown increases, can be identified as 6-ML CdS. PL excitation spectroscopy further confirms the successful formation of the heterostructure. The spectra acquired closely resemble the corresponding absorption spectra (Figure S2a–c in the Supporting Information), indicating the structural and optical consistency of the synthesized heterostructures.

**Figure 2:**
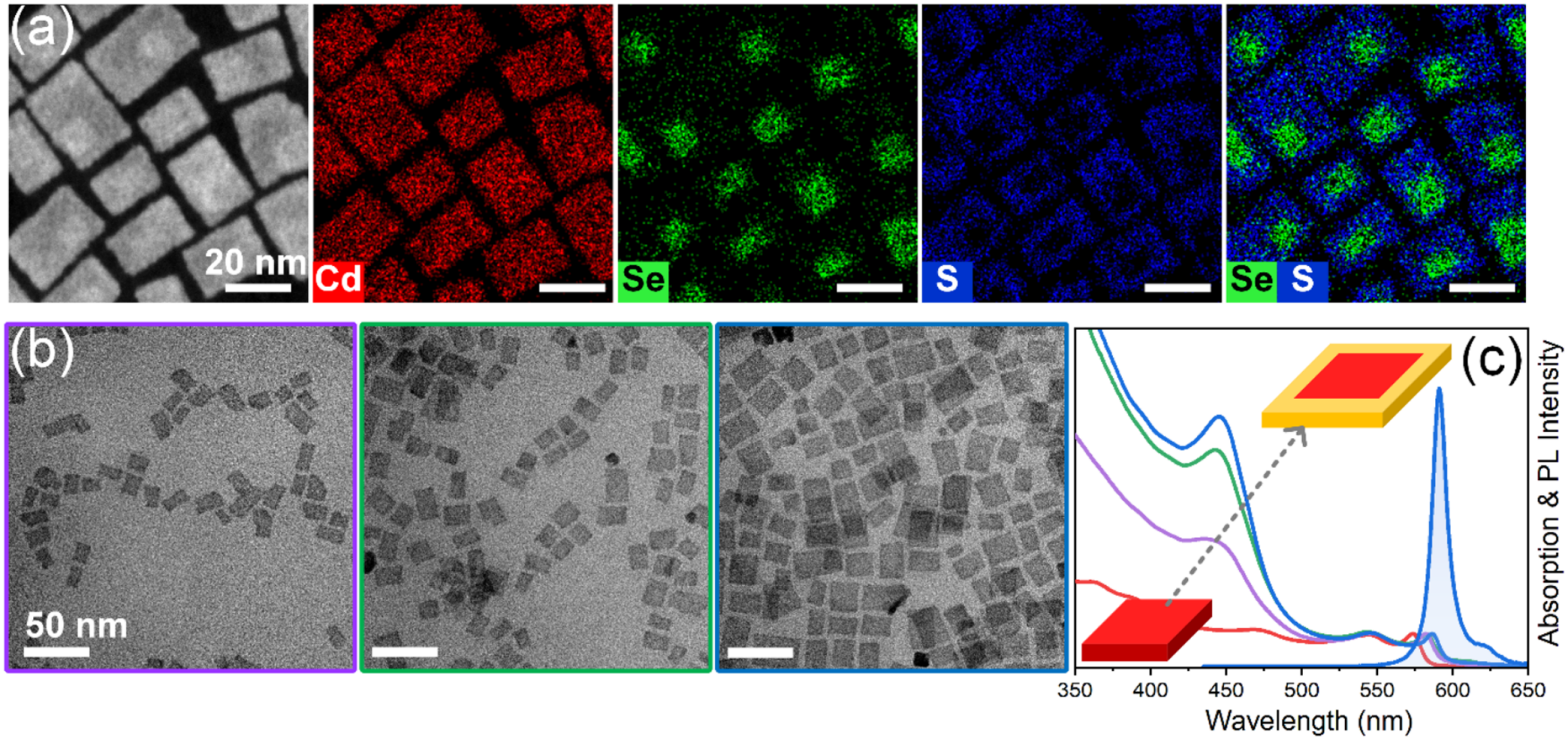
HAADF-STEM image and corresponding EDXS-based element distribution maps of 20 nm × 40 nm 6-ML CdSe/CdS core/crown NPLs (a). TEM images of 6-ML CdSe/CdS core/crown NPLs with different crown sizes (b) and corresponding absorption spectra and PL spectrum of 20 nm × 40 nm 6-ML CdSe/CdS core/crown NPLs (c). Colors of the boxes around TEM images in (b) correspond to the colors of the spectra in (c).

To gain further insight into the structure of the synthesized NPLs, we performed HAADF-STEM imaging and spectrum imaging analysis based on EDXS. This analysis directly confirmed a homogeneous distribution of cadmium in all NPLs (Figure 2a). It is evident that the distribution of selenium and sulfur within individual NPLs is not uniform. The Se-rich regions correspond to the CdSe cores, while the S-rich regions delineate the CdS crowns. XRD analysis (Figure S3) of the NPLs before and after CdS crown growth confirms that the platelets retain their cubic crystal structure. Following the crown formation, the reflections in the XRD patterns are shifted to larger angles compared to pure CdSe. The shift in reflections and the presence of the new peak at 30.2° confirm the formation of the cubic zinc-blende CdS crown. As expected, crown growth improved the PLQY, increasing it from 34% for the core NPLs to 42% for the core/crown NPLs with a lateral size of 20 nm × 40 nm. Meanwhile, the PL decay curves revealed a much shorter average lifetime of 2.4 ns for 6-ML CdSe/CdS core/crown NPLs compared to 5.2 ns for the initial core NPLs (Figure S4a). The shortening can be associated with an increased lateral size of the NPLs, and their stacking.^49,50^ However, it has been observed that stacking should result in a decrease of PLQY, which is not consistent with our findings. Thus, the reasons behind this phenomenon are unclear yet and require further investigations, which are beyond the scope of this work.

The successful crown growth demonstrated here stands in contrast to earlier efforts by Meerbach et al. to grow CdS crowns on CdSe NPLs in the presence of CdF_2_ and other cadmium halides.^29^ We propose an explanation for these different outcomes based on our theoretical studies of halogen adsorption on CdSe NPL sidewalls. A key distinction in Meerbach’s work is the rectangular shape of their NPLs, whereas our NPLs are nearly square. High-resolution TEM (HRTEM) studies have established that square CdSe NPLs typically exhibit sidewalls with {110} crystallographic orientation, while rectangular CdSe NPLs expose {100} sidewalls, as schematized in Figure 3a.^1–3,30,51–53^ Thus, in Meerbach’s work the rectangular NPLs possessed {100} sidewalls, while our square NPLs should feature {110} edges.

**Figure 3:**
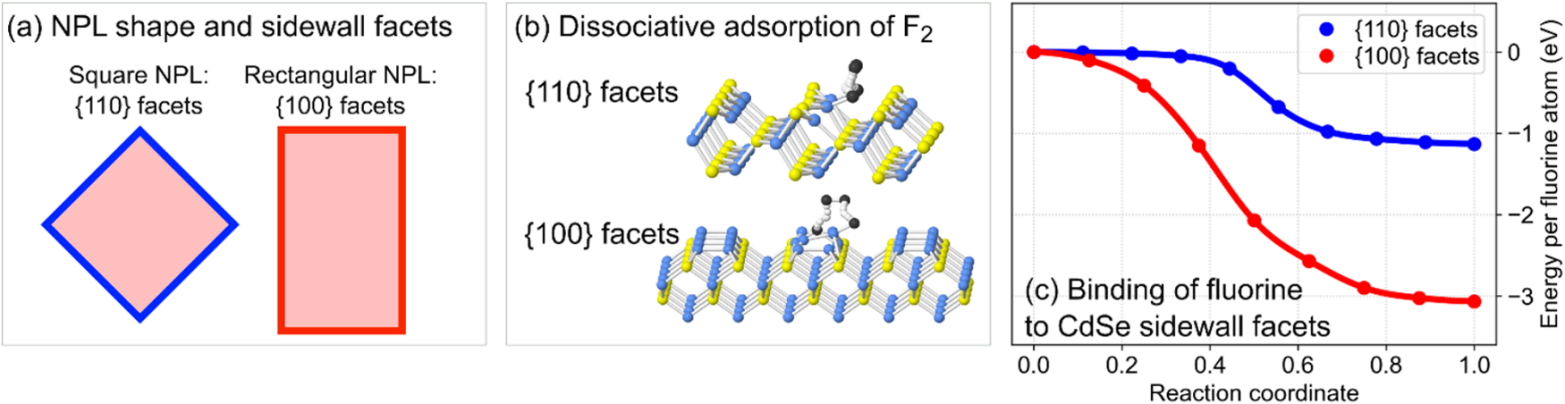
Relationship between NPL shape, sidewall facets, and the dissociative adsorption of fluorine F_2_ molecules on CdSe NPLs. (a) High-resolution TEM studies show that square NPLs are associated with {110} sidewall facets while rectangular NPLs are associated with {100} edges. (b) Reaction pathways for dissociative adsorption of F_2_ molecules on the {110} and {100} facets of CdSe, as determined by DFT calculations. (c) Potential energy surface along the reaction pathway, and the resulting binding energy of fluorine on {110} and {100} facets of CdSe.

To analyze the consequence of these shape differences for crown growth, we first simplify the CdF_2_ decomposition process and assume it yields Cd ions and stable F_2_ molecules. Our density-functional theory (DFT) calculations of F_2_ adsorption on CdSe reveal striking differences between the two types of facets. On both {110} and {100} facets, F_2_ undergoes dissociative adsorption without energy barriers, resulting in individual F atoms binding to surface Cd atoms (Figure 3b). However, the binding energies differ dramatically: 1 eV per F atom on {110} facets vs 3 eV on {100} facets, as shown in Figure 3c. This 3-fold stronger F–Cd binding on CdSe{100} effectively poisons these facets against further growth and could prevent crown formation on rectangular NPLs when CdF_2_ is present. Similar behavior occurs with other halides (CdCl_2_ and CdBr_2_), though with somewhat smaller binding energies (Table S1).

In order to corroborate our theory, we performed HRTEM analysis of the 6 ML NPLs (Figure 4a), the seeded-grown 6 ML NPLs (Figure 4b), and the 20 nm × 40 nm 6 ML CdSe/CdS core/crown NPLs (Figure 4c). It is noteworthy that all of the NPLs analyzed exhibit {100} sidewalls, despite their variation in shape, particularly between 6 ML NPLs, which appear squarer, and seeded-grown 6 ML NPLs, appearing rectangular. The available findings demonstrate the complexity of the crown formation and growth process. Further studies are required to establish a firm correlation between NPL shape, crystal orientation, and halide poisoning. We assume that the irregular shape of the side facets of 6 ML NPLs (Figure 4a) may offer enhanced accessibility for crown growth, as fluoride poisoning would not be as severe as is in the case of flat regular facets. This hypothesis, however, requires further investigations.

**Figure 4:**
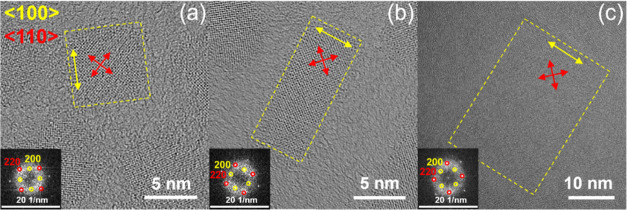
HRTEM images with corresponding FFT patterns (insets) of a single 6 ML CdSe NPL (a), a seeded-grown 6 ML CdSe NPL (b), and a 20 nm × 40 nm 6 ML CdSe/CdS core/crown NPL (c).

To grow a 7-ML core/crown CdSe/CdS NPLs heterostructure, we employed the same approach as for the 6-ML CdSe NPLs. However, our first attempt resulted in the formation of laterally enlarged 10 nm × 10 nm square NPLs (Figure 5b). The optical features of these NPLs closely resembled CdSe/CdS core/shell NPLs (Figure 5c) rather than core/crown structures. Specifically, the absorption and PL spectra of the heterostructure shifted significantly to longer wavelengths (hh transition from 591 to 645 nm, PL maximum from 593 to 650 nm). This behavior is not typical for the core/crown structures, where such shifts are generally minimal, as seen in the case of the 6-ML CdSe/CdS NPLs discussed above. Similar to the 6-ML CdSe/CdS core/crown NPLs, the element distribution maps (Figure 5a) show a homogeneous distribution of cadmium across the whole NPL. However, unlike the results shown in Figure 2a, the sulfur distribution was inhomogeneous, with a high concentration at the edges of the NPLs and some overlap with selenium regions. This suggests the formation of a thin CdS shell, yielding a core/crown/shell structure rather than a pure core/crown configuration. The work of Schlosser et al. demonstrated a correlation between the injection rate of the S-precursor and the onset of 3D overgrowth instead of 2D growth, leading to the formation of a shell instead of a crown.^45^ To address this we increased the injection rate in an attempt to achieve the desired core/crown structure. Although some NPLs were observed in the final product (Figure S5), the presence of significant byproducts—such as 3-ML CdS NPLs and cuboid-shaped particles—prevented quantitative isolation and accurate identification of 7-ML CdSe/CdS core/crown NPLs.

**Figure 5:**
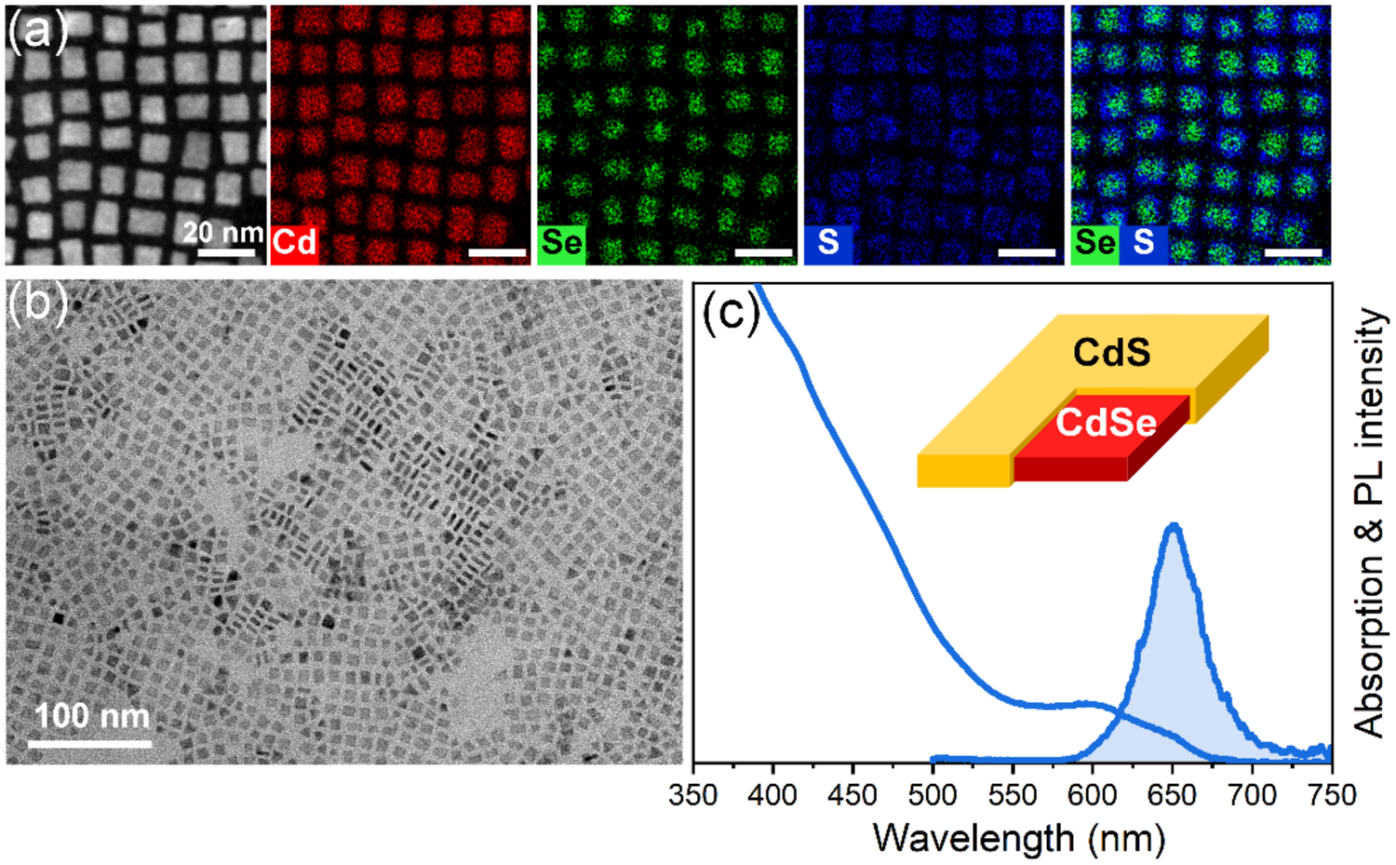
HAADF-STEM image and corresponding EDXS-based element distribution maps of 10 nm × 10 nm 7-ML CdSe/CdS core/shell NPLs (a). TEM image of 7-ML CdSe NPLs after attempted seeded growth resulting in the shell formation (b) with corresponding absorption and PL spectra (c).

To achieve the 2D crown growth, we modified the reaction conditions by removing TOP, which we suspected to be the primary cause of 3D overgrowth and byproduct particle formation. As hypothesized, the injection of sulfur dissolved in ODE enabled the formation of 7-ML CdSe/CdS core/crown NPLs, confirming our conjecture. During this crown growth, we also changed the source of cadmium precursor from cadmium/oleic acid/acetate prepared already in the particle dispersion, to cadmium/2-ethylhexanoic acid added with S-in-ODE solution via a syringe pump. This ensured a controlled and simultaneous addition of both sulfur and cadmium precursors. TEM analysis of the product revealed NPLs with an increased lateral size of up to 10 nm × 15 nm, and STEM-EDXS analysis proved the growth of the CdS crown (Figure 6a,b). The optical spectroscopy results shown in Figure 6c allow us to assign an absorption shoulder at about 460 nm in the heterostructure to the 7-ML CdS crown. This feature is less sharp than in thinner core/crown NPLs, likely due to weaker thickness confinement. Additionally, the small crown size may explain the absence of a particularly sharp CdS absorption feature, which could also be attributed to the formation of an alloyed CdSeS heterostructured crown. Alloyed crowns typically display less pronounced absorption signatures compared to pure CdS crowns.^49^ This interpretation was confirmed by a more pronounced absorption feature of a larger CdS crown (Figure S6a), where evidence of a thin CdS shell in this sample suggested a core/crown/shell heterostructure. After crown growth, the hh transition shifted from 594 to 606 nm, whereas the PL peak shifted from 596 to 608 nm. The PL excitation spectrum of the NPLs shown in Figure S2d closely resembled the corresponding absorption spectrum, confirming the absence of the secondary nucleation or growth of CdS NPLs or nanoparticles. This confirmed the successful formation of the core/crown heterostructure. Similar optical trends were observed using the seeded growth method under the same reaction conditions using Se-in-ODE solution as the selenium precursor. Despite the clear increase in lateral sizes, the optical features of the sample revealed byproducts, including some CdSe shell growth (Figure S7). As observed with 6-ML NPLs, crown growth on 7-ML NPLs significantly improved the PL intensity, increasing the QY from 47 to 72%. This improvement was accompanied by changes in PL decay time from 6.8 ns for the core NPLs to 4.3 ns for the heterostructure (Figure S4b), and similar changes in XRD diffraction patterns as for 6 ML CdSe/CdS core/crown NPLs (Figure S3).

**Figure 6:**
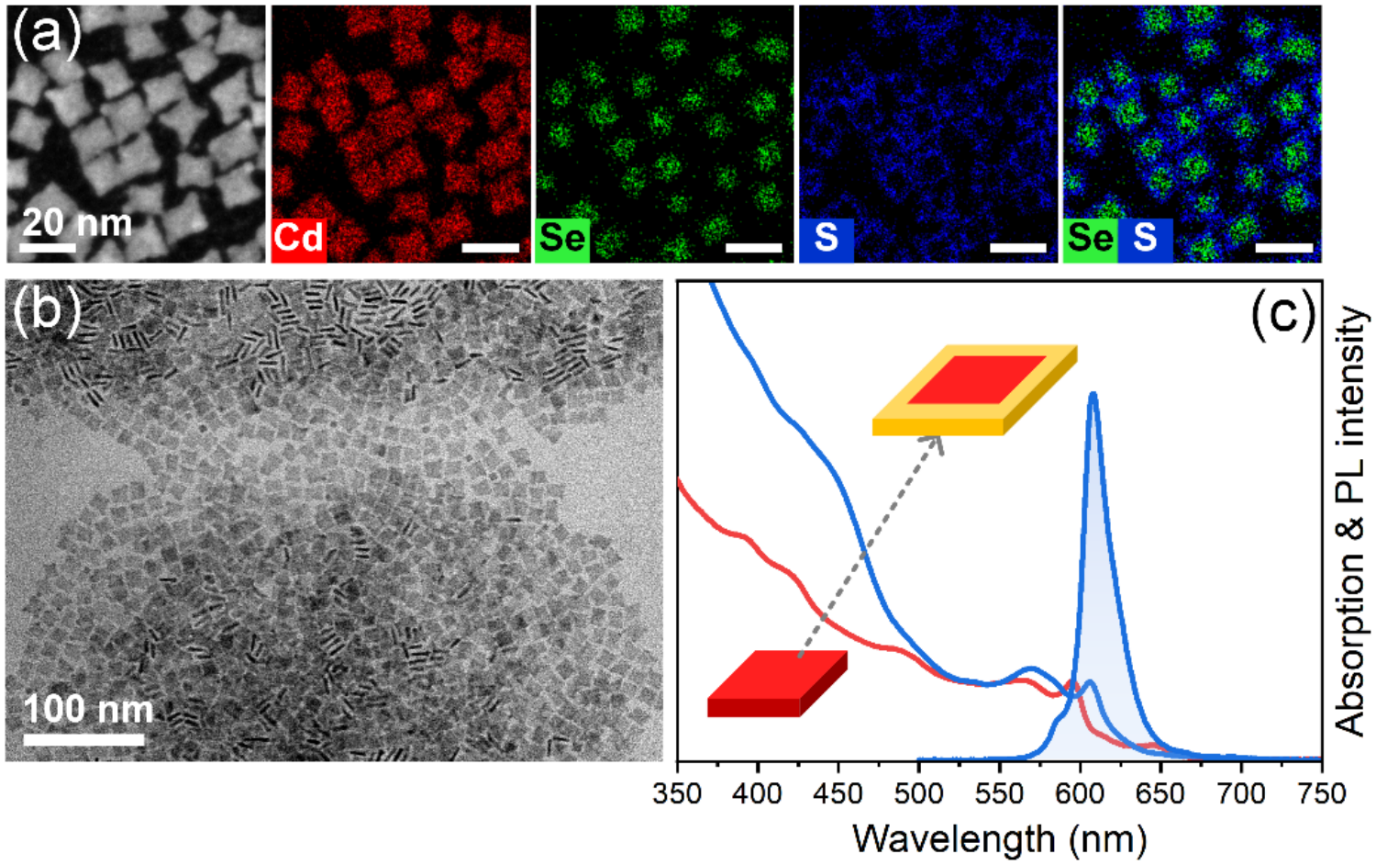
HAADF-STEM image and corresponding EDXS-based element distribution maps of 7-ML CdSe/CdS core/crown NPLs (a). TEM image of 7-ML CdSe/CdS core/crown NPLs (b) with corresponding absorption and PL spectra (c).

## Conclusions

We demonstrate novel approaches for the selective direct synthesis of 6- and 7-ML CdSe NPLs and the growth of related core/crown heterostructures. Theoretical calculations highlight the strong facet dependence of the fluoride binding energies and reveal that both the ligands and the shape of the NPLs play crucial roles in the heterostructure formation. The formation of these CdSe/CdS core/crown NPLs was confirmed by both optical and element distribution analysis. The obtained heterostructured NPLs exhibit narrow and intense PL at ca. 591 and 608 nm for 6- and 7 ML-thick NPLs, respectively, with a QY of up to 72% for 7-ML CdSe/CdS core/crown NPLs. By broadening the available spectral range of CdSe-based NPLs, we not only move closer to achieving exceptionally narrow, red-emitting nanocrystals, but also gain a deeper understanding of the formation mechanisms of thick NPLs and their heterostructures. We believe this progress extends beyond CdSe/CdS systems and may apply to other heterostructred nanocrystals.

## Supplementary Material



## Notes

Theoretical section; table of calculated binding energies per halogen atom on the CdSe(110) vs CdSe(100) surfaces; PLE spectra of 6- and 7-ML CdSe/CdS core/crown NPLs; XRD patterns and PL decay curves of 6- and 7-ML core CdSe and core/crown CdSe/CdS NPLs; additional TEM images of 7-ML CdSe NPLs; absorption and PL spectra, TEM and HAADF-STEM images, and corresponding EDXS-based element distribution maps of 7-ML CdSe/CdS core/crown/shell NPLs; TEM image of larger seeded-grown 7-ML CdSe NPLs with corresponding absorption and PL spectra (PDF)
