# Supplementary material for: Expanding the Family of Heterostructured CdSe/CdS Core/Crown Nanoplatelets: Six- and Seven-Monolayers-Thick Species
Source: Chem Mater. 2025 Aug 7;37(16):6151–60. doi: 10.1021/acs.chemmater.5c00650 (PMC12392805; doi:10.1021/acs.chemmater.5c00650)
Supplement: Supplementary file 1 [file cm5c00650_si_001.pdf]

SUPPORTING INFORMATION

Expanding the Family of Heterostructured CdSe/CdS Core/Crown Nanoplatelets: Six- and Seven-Monolayers-Thick Species

Volodymyr Shamraienko,[†] Valeriia Haidei,[†] Artsiom Antanovich,[†,‡] René Hübner,[§] Steven C. Erwin,[ǂ,] Vladimir Lesnyak,[†,*] and Alexander Eychmüller[†]*

[†]Physical Chemistry, TU Dresden, Zellescher Weg 19, 01069 Dresden, Germany

[‡]Institute of Physical Chemistry and Electrochemistry, Leibniz University Hannover, Callinstraße 3A, 30167 Hannover, Germany

[§]Institute of Ion Beam Physics and Materials Research, Helmholtz-Zentrum Dresden-Rossendorf e.V., Bautzner Landstrasse 400, 01328 Dresden, Germany

[ǂ]Center for Computational Materials Science, Naval Research Laboratory, 20375 Washington DC, United States

THEORETICAL SECTION

Density-functional theory calculations: We used density-functional theory (DFT) to determine the minimum-energy reaction pathway for dissociative adsorption of fluorine F_2 molecules on CdSe (100) and (110) surface, using projector-augmented-wave potentials as implemented in VASP.[1,2] The calculations were performed within the generalized-gradient approximation of Perdew, Burke, and Ernzerhof (PBE) to DFT.[3] CdSe NPLs grown in colloidal solution have the zinc-blende crystal structure and exhibit Cd-terminated (100) surfaces, while the (110) surfaces have a mixed Cd-Se termination. In the interest of simplicity we did not include ligands but instead considered adsorption onto the most energetically stable and fully relaxed bare surface: on CdSe(100) the Cd terminating layer forms Cd-Cd dimers, while the CdSe(110) surface is not reconstructed but the surface Cd-Se dimers are strongly tilted. We considered several possible adsorption pathways and found several to be energetically downhill with no activation barrier for adsorption. In these cases, the F_2 molecule splits spontaneously into two F atoms, which then adsorb by binding to surface Cd atoms as shown in Figure 3 in the main text. The reaction pathways and corresponding potential energy surfaces shown there were determined using the nudged elastic band method.[4] The most physically consequential results from these calculations are the binding energies on CdSe(110) versus CdSe(100) (per fluorine atom and referenced to the intact molecule far from the surface). These are summarized in Table S1 for the three halogens: fluorine, chlorine, and bromine. All three show strong binding only to the CdSe(100) surface, and weak or no binding to the CdSe(110) surface.

Table S1. Binding energy (eV) per halogen atom on the CdSe(110) vs. CdSe(100) surfaces, as determined from DFT.

**Halogen**	**CdSe(110), eV**	**CdSe(100), eV**
Fluorine	1.2	3.1
Chlorine	0.1	2.0
Bromine	0.0	1.8

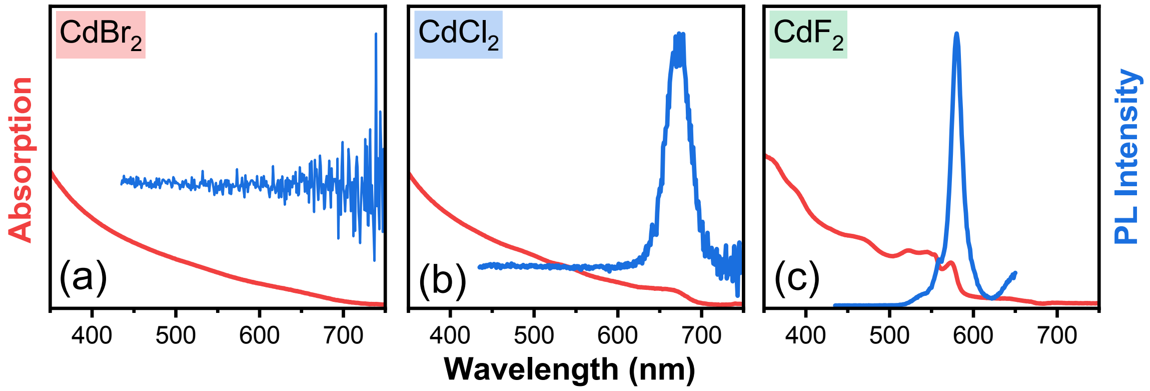

Figure S1. Absorption and PL spectra of the products after the first cleaning step (centrifugation of a crude mixture without addition of an antisolvent) after the attempt to synthesize 6-ML CdSe NPLs with different halides: (a) $CdBr_2$, (b) $CdCl_2$, and (c) CdF_2. Optical features of the NPLs can be seen only in the case of fluoride assisted synthesis.

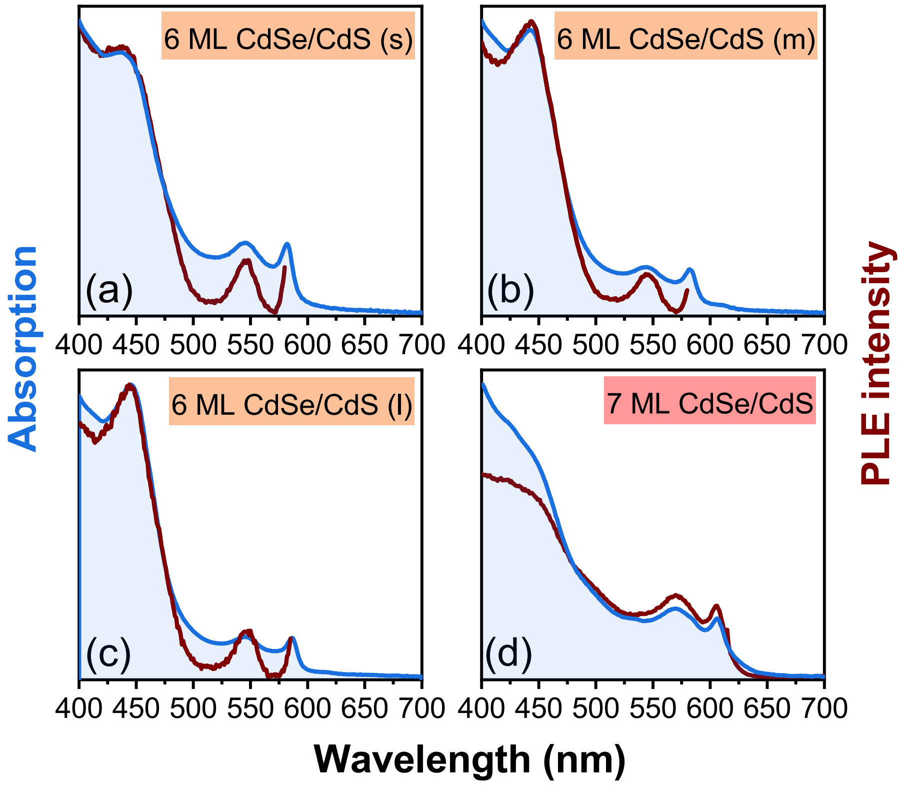

Figure S2. Absorption and PL excitation (PLE) spectra of 6- (a-c) and 7-ML (d) CdSe/CdS core/crown NPLs. Three differently sized CdS crowns were grown on 6-ML CdSe NPLs: small (a), medium (b), and large (c). One can clearly see an increasing contribution of CdS absorption upon the

crown size increase from (a) to (c). PLE spectra were acquired at 590 nm for 6-ML and at 615 nm for 7-ML CdSe/CdS core/crown NPLs.

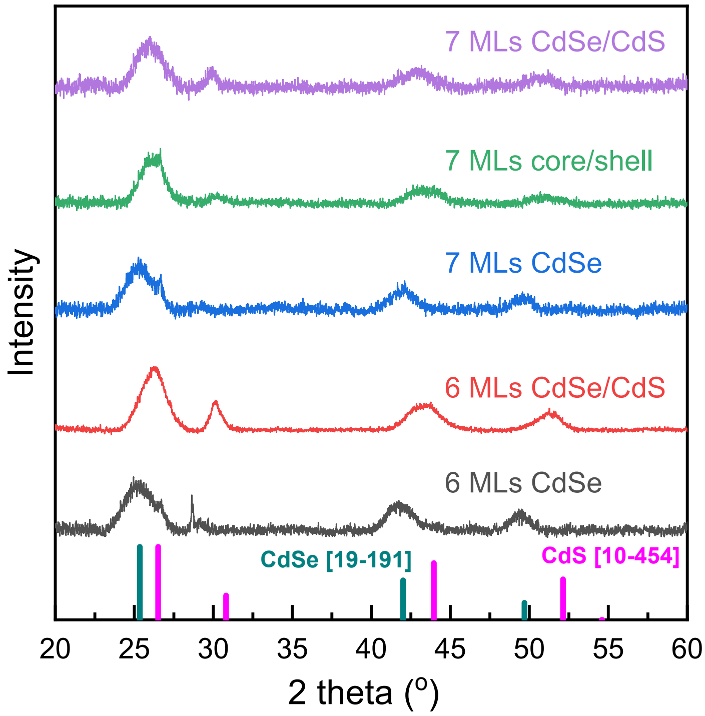

Figure S3. XRD patterns of 6- and 7-ML CdSe NPLs and their heterostructures.

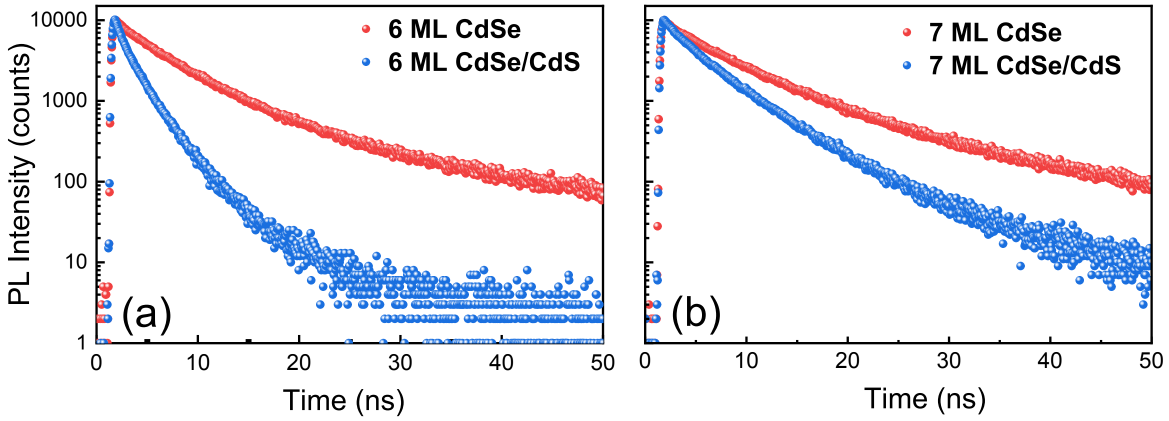

Figure S4. PL decay curves of 6- (a) and 7-ML (b) CdSe and CdSe/CdS core/crown NPLs.

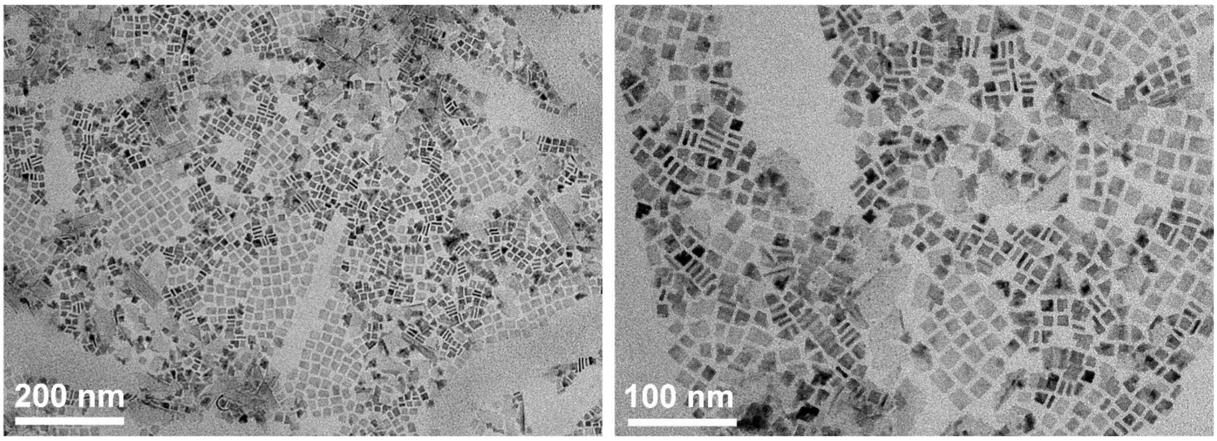

Figure S5. TEM images of 7-ML CdSe NPLs with various byproducts after the CdS crown growth using the method for the 6-ML CdSe/CdS NPLs with fast (12 mL/h) addition of S-in-TOP.

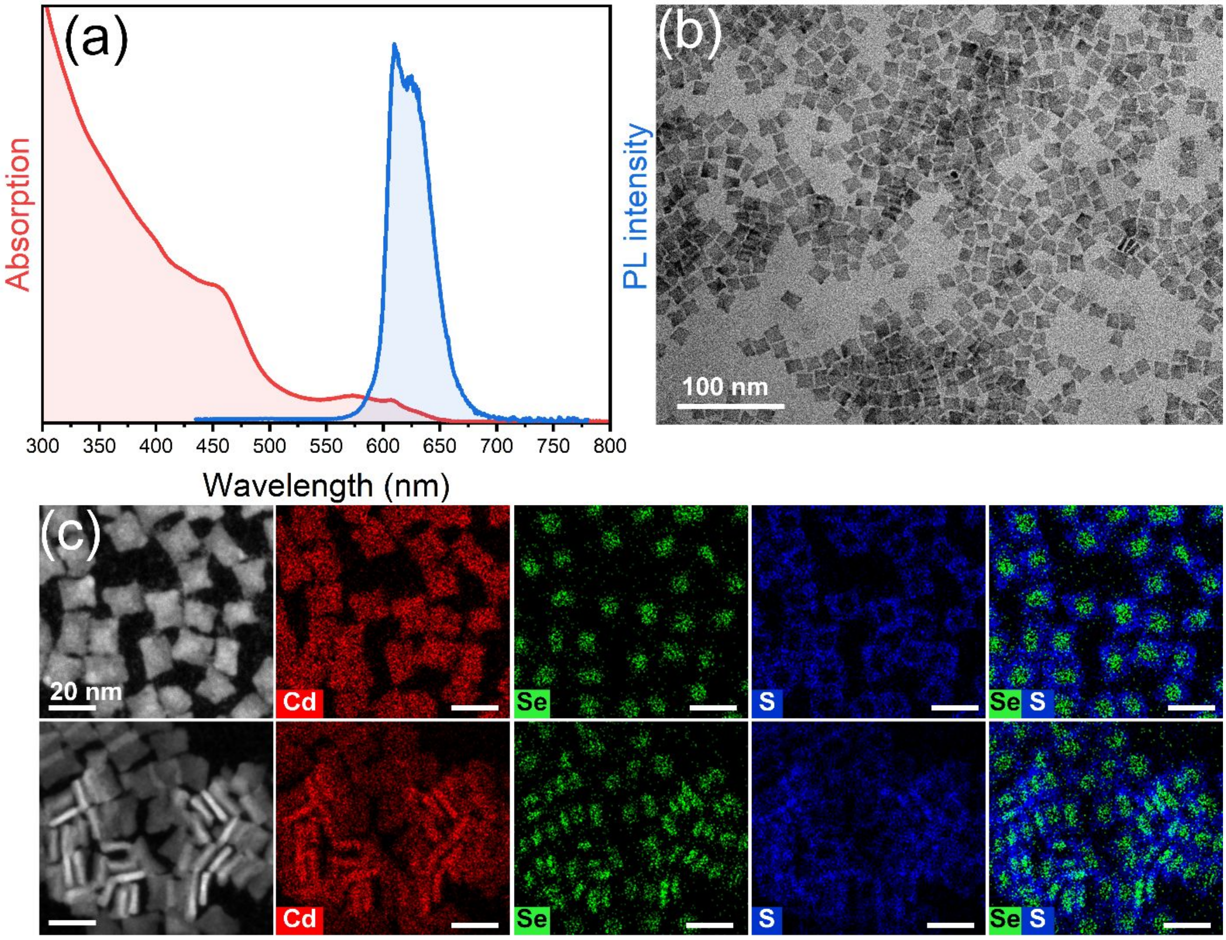

Figure S6. Absorption and PL spectra (a) of 7-ML CdSe/CdS core/crown/shell NPLs (a) obtained upon adding S-in-ODE at a rate of 3 mL/h with corresponding TEM image (b). HAADF-STEM images and corresponding EDXS-based element distribution maps of 7-ML CdSe/CdS core/crown/shell NPLs (c).

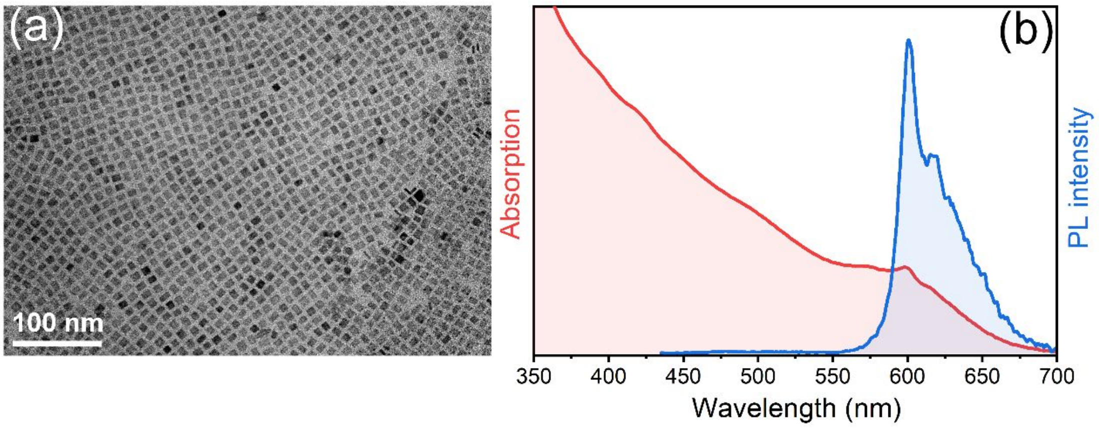

Figure S7. TEM image of larger 7-ML CdSe NPLs seeded-grown with Se-in-ODE (a) with corresponding absorption and PL spectra (b).

REFERENCES

(1) Kresse, G.; Furthmüller, J. Efficiency of Ab-Initio Total Energy Calculations for Metals and Semiconductors Using a Plane-Wave Basis Set. *Comput. Mater. Sci.* **1996**, *6* (1), 15–50. https://doi.org/10.1016/0927-0256(96)00008-0.

(2) Blöchl, P. E. Projector Augmented-Wave Method. *Phys. Rev. B* **1994**, *50* (24), 17953–17979. https://doi.org/10.1103/PhysRevB.50.17953.

(3) Perdew, J. P.; Burke, K.; Ernzerhof, M. Generalized Gradient Approximation Made Simple. *Phys. Rev. Lett.* **1996**, *77* (18), 3865–3868. https://doi.org/10.1103/PhysRevLett.77.3865.

(4) Henkelman, G.; Uberuaga, B. P.; Jónsson, H. Climbing Image Nudged Elastic Band Method for Finding Saddle Points and Minimum Energy Paths. *J. Chem. Phys.* **2000**, *113*, 9901–9904. https://doi.org/10.1063/1.1329672.
